# Filler-Induced Interstitial Nephritis in a Male-to-Female Transgender Person: A Case Report

**DOI:** 10.7759/cureus.31407

**Published:** 2022-11-12

**Authors:** Mohammad H Al-Thnaibat, Ahmed B Mohd, Omar B Mohd, Reem A Ghannam, Mohammad A Issa, Seri S Sawaqed

**Affiliations:** 1 Nephrology, Hashemite University, Zarqa, JOR; 2 Medicine, Hashemite University, Zarqa, JOR; 3 Clinical Sciences, Hashemite University, Zarqa, JOR; 4 Infectious Disease, Hashemite University, Zarqa, JOR

**Keywords:** case report, transgender, acute kidney injury, kidney biopsy, acute interstitial nephritis

## Abstract

Transgender individuals experience lower overall health outcomes than cisgender individuals due to a higher burden of chronic illnesses in this demographic. An early loss in renal function is frequently seen in acute interstitial nephritis (AIN), which is defined by the presence of inflammatory infiltrates and edema inside the interstitium. Infections or medication use can cause interstitial nephritis. In two-thirds of cases, interstitial nephritis caused by drugs is detected. Few people are affected by it, thus, it's thought to be immune-mediated rather than dose-dependent. In this report, a 32-year-old transgender female was admitted to a hospital due to generalized swelling following filler injections in the buttocks region.

It is important for doctors and patients to be informed about these procedures' potential risks. Additionally, more study has to be done on the negative effects of filler injections.

## Introduction

Transgender patients with chronic renal disease are receiving more and more care from nephrologists. However, they might not be as familiar with this patient population's unique problems [1]. When compared to the cisgender population, transgender people have a higher burden of chronic diseases, and their overall health outcomes are worse for a variety of reasons [2]. Due to coexisting mental health problems, which make them prone to substance addiction and dependency, and self-harm behaviors [‎2]. Numerous of these elements, such as non-adherence, HIV, highly active antiretroviral therapy, illegal substances, reno-vascular illness, and hormone therapy for gender affirmation, might affect or contribute to the underlying chronic kidney disease (CKD) [2]. Furthermore, exogenous hormone therapy is frequently used by transgender people to align their appearance more strongly with their gender identity. These therapies might alter their renal health and disease prevalence [3]. Transgender people have a degree of hypogonadotropic hypogonadism and decreased levels of endogenous sex hormones; therefore, they might need to use less exogenous sex hormones [‎2-‎3]. The risk of venous thromboembolism and cardiovascular illness is raised by exogenous estradiol medication and may contribute to chronic kidney disease [3]. Uncertainty exists regarding the effect of gender-affirming hormone therapy on the course of the glomerular filtration rate (GFR). Transgender patients' body composition and lean body mass are altered by gender-affirming hormone therapy using testosterone, estradiol, and anti-androgen medications. These changes have an impact on creatinine production and the accuracy of glomerular filtration rate (eGFR) equations [3].

Acute interstitial nephritis (AIN) is a renal condition characterized by the presence of inflammatory infiltrates and edema within the interstitium, which is typically accompanied by an immediate decline in renal function [4]. About 15% of the lesions in acute renal failure and up to 25% of the lesions in chronic renal failure are caused by interstitial nephritis, which frequently develops as a primary process [5]. Furthermore, interstitial nephritis can develop as a result of glomerular or vascular damage. Therefore, interstitial nephritis, the most prevalent and important lesion in nephrology, represents the last common pathway to all types of end-stage renal disease [5].

The aim of this report is to describe a rare case of filler-induced interstitial nephritis.

## Case presentation

A 32-year-old male-to-female transgender patient was referred to the nephrology department on 2/10/2022 for generalized edema and acute kidney injury (AKI) (Cr=2100 µmol/L). The patient was doing well until two weeks prior to admission. She complained of diffuse swelling all over her body which happened one day after filler injections which took place on 20/9/2022. The filler injections were two 500 ccs on every side of her buttocks region. One day following the injections, she had two episodes of dark black urine which were followed by anuria. In addition, progressive generalized swelling began.

Following this event, she began to worry and headed to a health clinic, where she was given diuretics. She did not improve and still had anuria despite taking the prescribed medication. She decided to head to another health clinic. At this clinic, an ultrasound was performed, which demonstrated increased echogenicity with normal-sized kidneys. Blood tests were also performed, and creatinine levels were found to be extremely elevated (Cr=2100 µmol/L). The patient was referred to the nephrology department of the hospital for further evaluation and hemodialysis. On 3/10/2022, the patient was admitted to the hospital.

**Figure 1:**
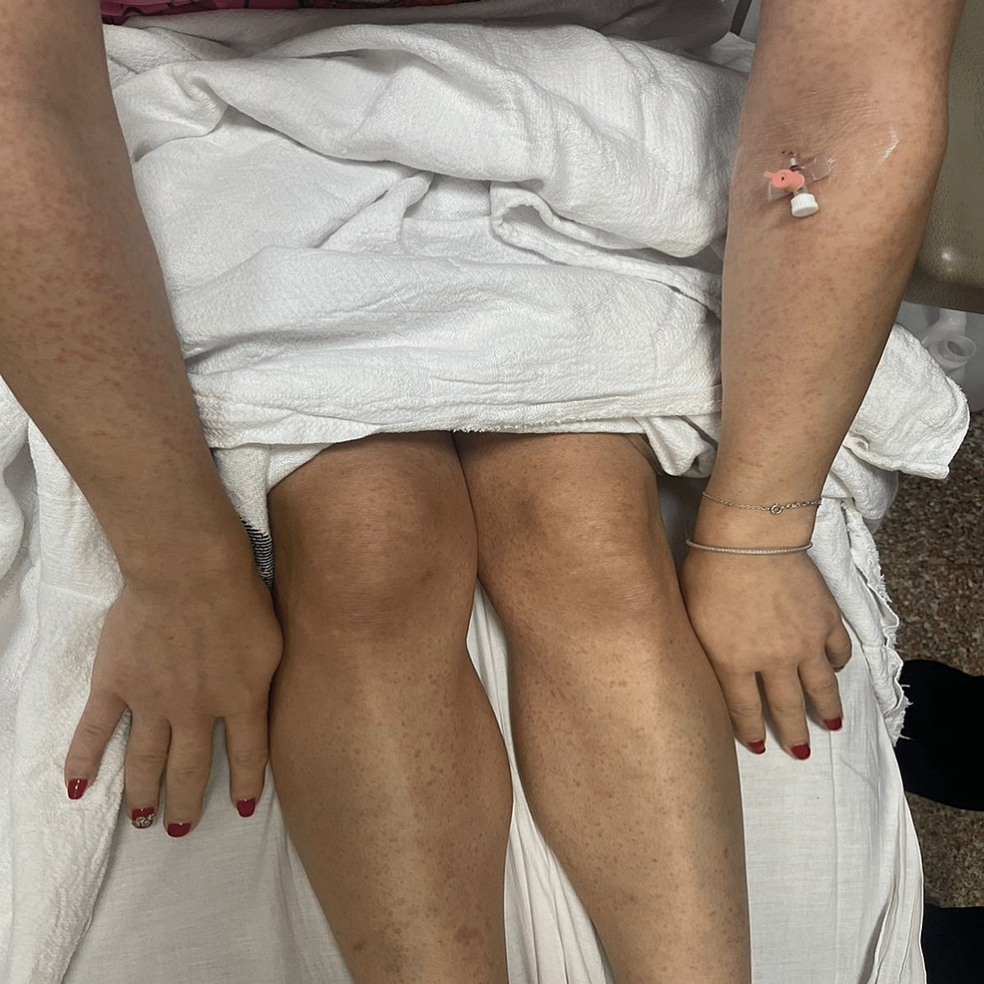
Generalized rash the patient developed two days after the filler injection

On review of systems, she had postural hypotension, decreased level of consciousness, weakness, dizziness, and loss of appetite. Also, she had hoarseness of voice, constipation, dysphagia, and heartburn. Furthermore, the patient had a generalized rash, as demonstrated in Figure 1. All of them happened two days after the filler injection.

The patient is an alcoholic. She consumed five to six cups daily for four months prior to admission. She has multiple male sexual partners and always has protected intercourse. She is HIV-negative.

Her history included hormone replacement therapy for two months at the age of 16, which was discontinued, breast augmentation at age 19, nose job at age 24, Brazilian butt lift (BBL) at age 27, and gastric sleeve at age 31, all without complications. She has not taken any vaccinations except for two shots of the BioNTech Pfizer vaccine. She has been previously addicted to multiple drugs, such as pregabalin, weed, cocaine, methamine, alprazolam, and amphetamine. She has been off the drugs since her therapy at a rehab center at the age of 30.

On admission, the patient's condition was stable, but her blood tests were worrying. The patient had metabolic acidosis with respiratory compensation (PH= 7.314, HCO3= 15.1, PaCO2= 24.7). In addition, creatinine levels were elevated (Cr=2100). Table 1 demonstrates the results of her blood tests on admission. Furthermore, her urinalysis results are shown in Table 2.

**Table 1:** Results of the patient's blood tests at the time of admission Hgb - hemoglobin

Blood test	Patient value	Normal value
Glucose	6.01 mmol/L	3.9-6.1
Urea	44.0 mmol/L	2.14-7.14
Sodium	130 mmol/L	135-152
Potassium	4.55 mmol/L	3.5-5.3
Creatinine	2021.80 µmol/L	66.4-119.3 µmol/L
PH	7.314	7.35-7.45
PaO2	106 mmHG	80-100 mmHG
PCO2	24.7mmHG	35-45 mmHG
HCO3	15.1 mmol/L	22-28 mmol/L
Total CO2	24.7mmol/L	25-30 mmol/L
Hgb	11.5 gm/dl	
O2 saturation	98.5	97-100

**Table 2:** The patient's urinalysis results at the time of admission

Test name	Patients value	Normal value
RBC	20-50 H /HPF	0-3
WBC	0-5 H/HPF	0-4
Urobilinogen	Negative	Negative
Ketones	Negative	Negative
Glucose	Negative	Negative
Protein	Trace 10 mg/dl	Negative
PH	6.0	
Specific gravity	1.030	1.005-1.030
Color	Yellow	
Character	Clear	

Urgent hemodialysis was ordered for fluid overload and uremic encephalopathy. Following the first hemodialysis procedure, the patient's creatinine levels improved (Cr=1639.6). Methylprednisolone 1-gram IV was given for three days, and chlorpheniramine maleate 10 mg IM was also given for three days. Furthermore, the second hemodialysis procedure also reduced the Cr levels (Cr=1405.7).

**Figure 2:**
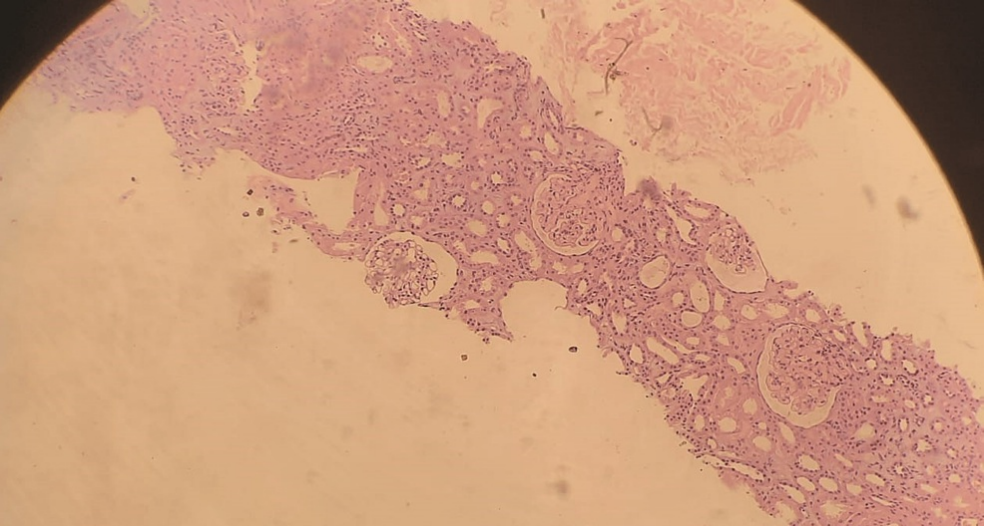
Cellular infiltration on a low-power field

**Figure 3:**
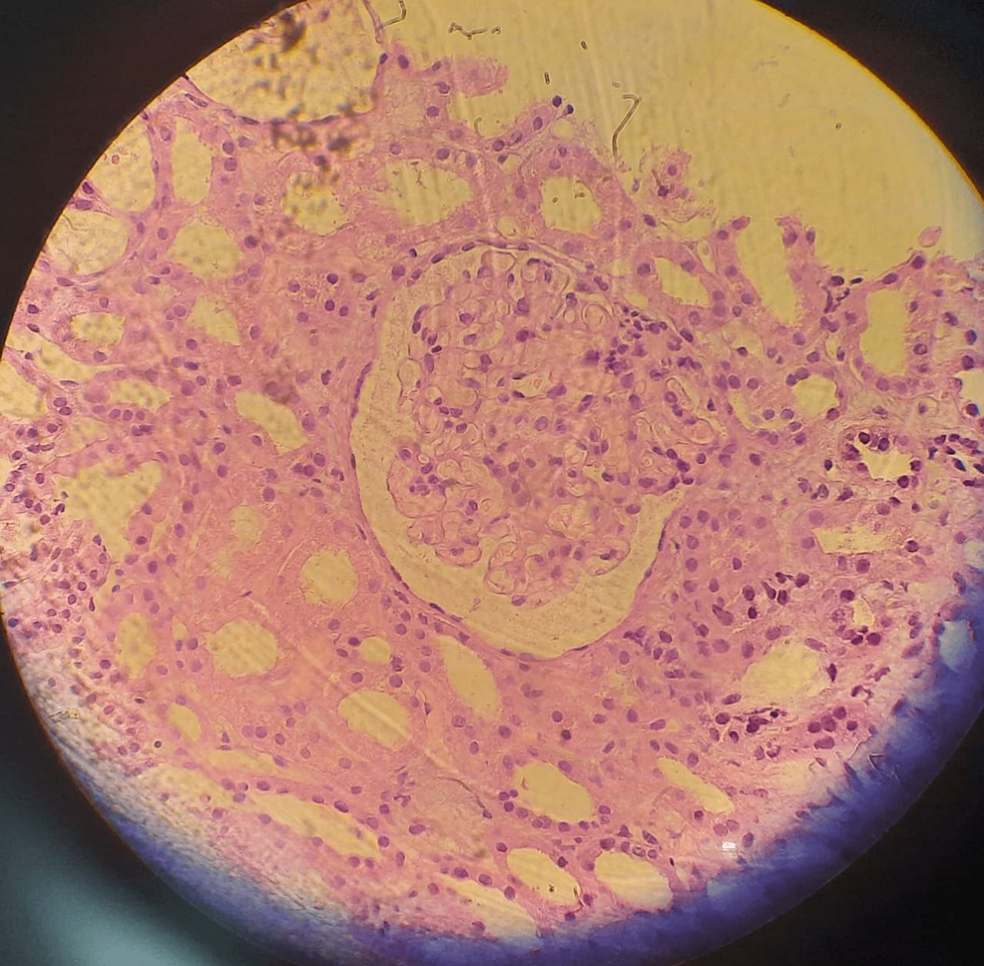
Kidney biopsy composed of cortex and medulla This demonstrates very minimal mesangial cell proliferation and an increase in the mesangial matrix. No crescents or thrombosis are seen. Some protein and RBC casts are seen in the renal tubules. The interstitium demonstrates mostly mononuclear inflammation with the number of interstitial eosinophils.

The patient was sent to the interventional radiology unit for a kidney biopsy. A left renal biopsy under ultrasound guidance was performed without any complications. The biopsy result demonstrated very minimal mesangial cell proliferation and an increase in the mesangial matrix. No crescents or thrombosis were seen. Some protein and RBC casts were detected in the renal tubules. The Interstitium demonstrated mostly mononuclear inflammation with a number of interstitial eosinophils. Immunofluorescence study detected IgA (IgA: +2). The patient was diagnosed with interstitial nephritis. Figure 2 demonstrates cellular infiltration on a low-power field. Figure 3 demonstrates the renal changes that were found on the biopsy. Also, Table 3 demonstrates the sequence of events of this case. 

**Table 3:** Sequence of the events

Date	Event
20/9/2022	Filler injection in the buttocks region
21/9/2022	Two episodes of black urine followed by anuria
21/9 - 2/10	Progressive generalized swelling; took diuretics without any improvement
2/10/2022	The patient visited a health clinic: ultrasound demonstrated bilateral hydronephrosis and abnormal shape of the kidneys; creatinine levels were found to be extremely elevated (Cr=2100 µmol/L); the patient was referred to the nephrology department of the hospital for further evaluation and hemodialysis
3/10/2022	The patient was admitted to the hospital; the first hemodialysis was done
6/10/2022	The second hemodialysis was done; biopsy was taken
7/10/2022	Biopsy results demonstrated interstitial nephritis

After 10 days of corticosteroid therapy (1 gram Methylprednisolone for three days, then the patient was switched to oral prednisone 60 mg a day), the patient's creatinine levels improved critically. Table 4 demonstrates the decline in creatinine levels of the patient after being compliant with the therapeutic regimen.

**Table 4:** Decline in creatinine levels of the patient after being compliant with the therapeutic regimen

Date	Specimen	Creatinine levels
19/10/2022	serum	95.50 H
13/10/2022	serum	230.80 H
12/10/2022	serum	287.53 H
12/10/2022	serum	368.60 H
11/10/2022	serum	559.60 H
10/10/2022	serum	772.60 H
9/10/2022	serum	962.30 H
8/10/2022	serum	1065.50 H
7/10/2022	serum	1013.60 H
6/10/2022	serum	1468.60 H
6/10/2022	serum	1479.70 H
5/10/2022	serum	1405.70 H
4/10/2022	serum	1639.60 H
3/10/2022	serum	2021.60 H

## Discussion

This case report discusses a 32-year-old male-to-female transgender patient who was administered to the hospital after receiving filler injections (two 500 ccs on every side of her buttocks region). The substance which the patient was injected with is unknown. The patient's renal function severely declined, and she was later diagnosed with interstitial nephritis after performing a renal biopsy.

Interstitial nephritis is a renal disease that is characterized by the presence of both diffuse and patchy inflammatory cell infiltrates, with a common interstitial edema finding, which is typically accompanied by a decline in renal function. Lymphocytes CD4 T cells are the most prevalent kind; macrophages, eosinophils, monocytes, neutrophils, and plasma cells make up most interstitial infiltrates [6].

Interstitial nephritis may be infection or drug-induced. Drug-induced interstitial nephritis is seen in two-thirds of the cases. Since it only affects a few individuals, it's believed to be immune-mediated rather than dose-dependent. Moreover, T cells seen in the biopsy confirm that the reaction is a cell-mediated hypersensitivity reaction [7]. The immune system is stimulated either by a drug functioning as an external antigen or hapten or by molecular mimicry to one of the tubular antigens. Drugs like lidocaine, sulfamethoxazole, carbamazepine, and radiocontrast agents have been examples of drug-induced interstitial nephritis [7]. It is believed that the filler's unknown components elicited drug-induced interstitial nephritis in our patient.

IgA, however, was detected by light microscopy and Immunofluorescence. This is probably an accidental finding not related to our patient's symptoms. In a study including kidney donors, zero-hour allograft biopsies were performed on 510 allografts (446 from living donors and 64 from cadaveric donors). Mesangial IgA deposition was present in 82 of the 510 allografts. This indicates that mesangial immunoglobulin A (IgA) deposition could be found in asymptomatic patients [8].

Corticosteroid therapy was administered to the patient due to the evidence that early corticosteroid therapy has been associated with rapid and complete recovery for patients diagnosed with drug-induced acute interstitial nephritis [9]. A retrospective study including 61 patients with biopsy-proven drug-induced acute interstitial nephritis discovered a significant correlation between the delay in the onset of steroids and serum creatinine levels, and that an interval longer than seven days between drug withdrawal and the onset of steroid treatment was the only clinical factor that significantly increased the risk of an incomplete recovery of renal function [9]. As a result, the study strongly suggests that steroid treatment is indicated in drug-induced interstitial nephritis and should be initiated as soon as possible after diagnosis to avoid the risk of incomplete renal function recovery [9]. Therefore, our patient was administered corticosteroid treatment immediately.

A case investigation conducted by The North Carolina Division Of Public Health (NCDPH) with the cooperation of the CDC and the food and drug administration included three female patients who demonstrated acute kidney failure following filler injections [10]. One of the patients is a previously healthy 26-year-old woman who received the filler injections in the buttocks region over two sessions. The patient experienced symptoms one hour after the second session, including lightheadedness, abdominal pain, and nausea. After four days, the patient was administered, and hemodialysis was initiated. Following a renal biopsy, the patient was diagnosed with interstitial nephritis. The patient was discharged after two weeks, however, hemodialysis continued one week after discharge. After hemodialysis was discontinued, her serum levels returned to normal (Cr=95.0 µmol/L) [10].

In terms of similarities, both patients had severe renal impairment requiring hemodialysis and were diagnosed with interstitial nephritis after filler injections. However, the onset and form of symptoms vary amongst both patients. One day following the filler injection, our patient experienced black urine, anuria, widespread weakness, dizziness, and a lowered level of awareness. However, the patient mentioned in the NCDPH investigation experienced lightheadedness, stomach discomfort, and nausea one hour after her second filler session. One thing to take into consideration is that our patient is a transgender woman who had many operations prior to the filler injections. She also took hormone replacement therapy at the age of 16.

## Conclusions

Various cosmetic injections which contain exogenous material might cause severe adverse effects. Filler-induced interstitial nephritis is one of these serious complications. A biopsy and early recognition of the pathology could alter the course of the illness. Furthermore, corticosteroid administration is one of the most important therapeutic interventions, especially if given immediately.

Medical education and awareness of the possible complications of these procedures are necessary. Moreover, further research on the adverse effects of fillers and drugs must be done.
